# Integration of A Deep Learning Classifier with A Random Forest Approach for Predicting Malonylation Sites

**DOI:** 10.1016/j.gpb.2018.08.004

**Published:** 2019-01-11

**Authors:** Zhen Chen, Ningning He, Yu Huang, Wen Tao Qin, Xuhan Liu, Lei Li

**Affiliations:** 1 School of Basic Medicine, Qingdao University, Qingdao 266021, China; 2 School of Data Science and Software Engineering, Qingdao University, Qingdao 266021, China; 3 Department of Biochemistry, Schulich School of Medicine and Dentistry, University of Western Ontario, London, Ontario N6A 5C1, Canada; 4 Department of Information Technology, Beijing Oriental Yamei Gene Technology Institute Co. Ltd., Beijing 100078, China; 5 Qingdao Cancer Institute, Qingdao University, Qingdao 266021, China

**Keywords:** Deep learning, Recurrent neural network, LSTM, Malonylation, Random forest

## Abstract

As a newly-identified protein post-translational modification, **malonylation** is involved in a variety of biological functions. Recognizing malonylation sites in substrates represents an initial but crucial step in elucidating the molecular mechanisms underlying protein malonylation. In this study, we constructed a **deep learning** (DL) network classifier based on long short-term memory (**LSTM**) with word embedding (LSTM_WE_) for the prediction of mammalian malonylation sites. LSTM_WE_ performs better than traditional classifiers developed with common pre-defined feature encodings or a DL classifier based on LSTM with a one-hot vector. The performance of LSTM_WE_ is sensitive to the size of the training set, but this limitation can be overcome by integration with a traditional machine learning (ML) classifier. Accordingly, an integrated approach called LEMP was developed, which includes LSTM_WE_ and the **random forest** classifier with a novel encoding of enhanced amino acid content. LEMP performs not only better than the individual classifiers but also superior to the currently-available malonylation predictors. Additionally, it demonstrates a promising performance with a low false positive rate, which is highly useful in the prediction application. Overall, LEMP is a useful tool for easily identifying malonylation sites with high confidence. LEMP is available at http://www.bioinfogo.org/lemp.

## Introduction

Various protein post-translational modifications (PTMs), such as lysine ubiquitination and acetylation, are detected at lysine residues. Lysine malonylation (Kmal) is a newly identified PTM type that is evolutionarily conserved in both eukaryotic and prokaryotic cells [1]. Kmal is associated with various biological processes. For instance, malonylation on K184 of glyceraldehyde 3-phosphate dehydrogenase (GAPDH) regulates the activity of this key metabolic enzyme [2], whereas several Kmal sites in histone proteins have potential connections with cancer [3].

Although many efforts have been devoted to investigating the cellular mechanisms of Kmal, its biological significance remains poorly understood. To characterize malonylation at the molecular level, it is important to identify the Kmal sites of protein substrates [4]. Recent advances in high-throughput experimental techniques have identified thousands of Kmal-containing peptides [4], [5]. These data have strengthened the fundamental understanding of the sequence/structural characteristics of Kmal. However, due to the dynamic properties and low abundance of protein malonylation *in vivo* and the limitations of experimental investigations (*e.g.*, labor-intensive, time-consuming, and costly), identifying Kmal sites on a large scale remains an enormous challenge.

In tandem with the experimental identification of Kmal sites, there is an urgent need to predict Kmal sites computationally. Many predictors have been developed using feature selection strategies. For instance, site-modification network profile, site-specific modification profile, functional information of proteins, and the combination of multiple kernel support vector machines (SVM) were employed for the prediction of PTM sites [6], [7], [8], [9]. So far, two *in silico* programs have been developed for the prediction of Kmal sites. Mal-Lys is based on SVM incorporated with the features including protein sequence information, position-specific amino acid propensity, and physicochemical properties [10]. MaloPred is also based on SVM integration with the features from sequence information and evolutionarily-derived information [11]. Additionally, an SVM algorithm was developed for the prediction of multiple lysine modifications, including malonylation [12].

Aside from traditional machine learning (ML) methods (*e.g*., SVM), the deep learning (DL) model is an increasingly promising ML algorithm. DL has a strong capability for learning sparse representation in a self-taught manner with multiple hidden layers, whereas the conventional ML algorithms require experts to pre-define informative features [13]. With the acceleration of graphics processing units, DL has become more efficiently trained, and the scope of its application has therefore been dramatically expanded. DL has been applied in the field of bioinformatics to predict RNA-binding sites [14], protein secondary structures [15], protein disorders [16], protein phosphorylation sites [17], ubiquitination sites [18], and nitrosylation sites [19]. DL has also been extensively applied in the field of biomedicine [20].

In this study, we constructed an LSTM-based classifier with a word embedding approach, dubbed LSTM_WE_, for the prediction of Kmal sites. We focused on mammalian species because 98% of known Kmal sites were identified from humans and mice [21]. LSTM_WE_ outperformed the conventional ML classifiers with different pre-defined feature encodings using both cross-validation and an independent test. Furthermore, we developed a LSTM-based ensemble malonylation predictor, named LEMP, which integrates LSTM_WE_ and the random forest (RF) classifier with a novel encoding of enhanced amino acid content (EAAC). LEMP performed better than individual components as well as the currently available malonylation predictors. Overall, LEMP is a useful tool for identifying Kmal sites with high confidence.

## Methods

### Dataset construction

The Kmal peptides were derived from mice and humans in two proteomic assays [2], [22]. To construct a non-redundant dataset with high confidence, we referred to the procedure established by Chen et al. [23] and generated the datasets for training and test as follows (Figure S1). The 10,368 Kmal sites with high confidence (*i.e.*, Kmal peptides with Andromeda scores > 50 and localization probability > 0.75 [24]) were collected as positive sites, and the remaining lysine residues (142,830) on the Kmal-containing proteins were considered negative sites. (2) Malonylation-containing proteins with sequence identities greater than 30% using the CD-HIT tool [25] were clustered and aligned using ClustalW2 [26]. In every cluster, the protein with the highest number of Kmal sites was selected as the representative, in which lysine sites that were experimentallyverified to be malonylated were considered as positive sites and the remaining lysine sites were taken as negative sites. It should be noted that the lysine sites in the representative were not considered negative if the aligned counterparts from other members of the same cluster can be malonylated. In this step, the dataset contained 5359 positive sites and 92,980 negative sites from 2127 representatives. (3) For every site, we extracted 7-residue peptides (−3 to +3) with the lysine site in the center from the representatives. If the peptides containing positive sites (*i.e.*, positive peptides) were identical to the peptides containing negative sites (*i.e.*, negative peptides), both peptides were removed. As a result, 5288 positive peptides and 88,636 negative peptides were retained for further analyses. (4) To test the optimal sequence window for model construction, we set the sequence window to six different sizes (*i.e.*, 15, 19, 23, 27, 31, and 35) and compared their performance via a ten-fold cross-validation (Figure S2). The window size of 31 showed the largest area under receiver operating characteristic (ROC) curve (AUC) and was thus selected, which was consistent with the previous analysis of other modifications [23]. It should be noted that if the central lysine site was located near the N-terminus or C-terminus of a protein sequence, the gap symbol ‘-’ was assigned to fill in the corresponding positions to ensure that the peptides had the same window size. (5) The dataset was separated into two groups: one for cross-validation and the other for an independent test. The peptides from 4/5 of the Kmal-containing proteins (*i.e.*, 1702 proteins with 4242 positive peptides and 71,809 negative peptides) were subjected to ten-fold cross-validation, and the peptides from the remaining proteins (*i.e.*, 405 proteins including 1046 positive peptides and 16,827 negative peptides) were employed as the independent test dataset (Figure S1 and Table S1).

### Feature encodings and construction of classifiers

#### EAAC encoding

The AAC encoding that reflects the frequency of 20 amino acid residues surrounding the modification site has been widely used in the prediction of various types of PTM sites [11], [27]. Here, based on the AAC encoding, we designed an EAAC encoding scheme in which the frequency of the 20 amino acid residues was counted in the window continuously sliding from the N-terminus to C-terminus of each peptide in the dataset. The sliding window size was selected as 8 via ten-fold cross-validation (Figure S3). Therefore, a peptide with 31 residues corresponded to 24 (31−8 + 1) sliding windows and its vector dimension of the EAAC encoding was 24 × 20 (amino acids) = 480.

#### EAAC-encoding RF classifier RF_EAAC_

RF, as one of the ML methods, has been used in a variety of bioinformatics studies, demonstrating stable and effective performance [28], [29], [30], [31]. It integrates different decision trees and chooses the classification with the highest number of votes from the trees. Each tree depends on the values of a random vector sampled independently with the same distribution for all trees in the forest. The margin of error of RF depends on the strength of the individual trees in the forest and the correlation between them. The EAAC encoding was used as input to train the RF classifier, resulting in 1000 decision trees by randomly selecting $\sqrt{d}$ number of variables as its candidate (*d* is the dimension of input feature vector). The RF classifier was implemented using the Weka software package (Version 3.8.1).

#### AAindex encoding RF classifier RFAAindex

AAindex is a database of numerical indices representing various physicochemical and biochemical properties of amino acids and pairs of amino acids (http://www.genome.jp/aaindex/). We collected 544 physicochemical properties from the AAindex database and retained 531 properties after the removal of properties with “NA” in the amino acid indices. We calculated the performance for each property using the RF classifier described above using the ten-fold cross-validation dataset (Figure S1 and Table S1). We selected the top 11 properties with AUC > 0.7 (Table S2). Therefore, a peptide with 31 residues was converted to a vector of 341 (31 × 11) dimensions as the AAindex encoding. The construction of the RF_AAindex_ was the same as that of RF_EAAC_.

#### One-hot encoding

Each peptide with 31 residues was represented as a 31 × 20 matrix, in which each residue of the peptide is represented as a 20-dimensional vector filled with 19 zeros and a one in the index corresponding to the specific residue. When the left or right neighboring amino acid residues cannot fit the window size of 31, dashes ‘-’ are filled in these positions and encoded to 0.05 across the 20-dimensional vector [18].

### Integration of the classifiers

The prediction score *S* of LEMP was calculated by integrating the classifiers (LSTM_WE_ and RF_EAAC_) according to the following equation:

(1) $$\log\left(\frac{S}{1\;-\;S}\right)\;\;\text{=}\;\;\sum_{\text{i}=1}^{2}w_{i}C_{i}\;+\;b$$

where *b* means the bias, *w_i_* and *C*_i_ refer to the weight and output of the classifier *i*, respectively. The score *S* denotes the confidence level of the central lysine to be malonylated. *w_i_* and *b* were optimized with a ten-fold cross-validation using the logistic regression model based on the ‘glm’ function in the R package (http://www.r-project.org/).

### Performance assessment of the predictors

The performance of each predictor was assessed by ten-fold cross-validation and an independent test. Four measurements, *i.e.*, accuracy (Ac), sensitivity (Sn), specificity (Sp), and Matthew’s correlation coefficient (MCC), were adopted to evaluate the prediction performance. They were defined as follows:

(2) $$\mathrm{Ac}\;=\;\frac{\mathrm{TP}\;+\;TN}{\mathrm{TP}\;+\;FN\;+TN\;+\;FP}$$

(3) $$\mathrm{Sn}\;=\;\frac{\mathrm{TP}}{\mathrm{TP}\;+\;FN}$$

(4) $$\mathrm{Sp}\;=\;\frac{\mathrm{TN}}{\mathrm{TN}\;+\;FP}$$

(5) $$\mathrm{MCC}\;=\;\frac{\mathrm{TP}\;\times \;\mathrm{TN}\;-\;\mathrm{FP}\;\times \;\mathrm{FN}}{\sqrt{\left(TP\;+\;FP)\;\times \;(\mathrm{TP}\;+\;\mathrm{FN})\;\times \;(\mathrm{TN}\;+\;\mathrm{FN})\;\times \;(\mathrm{TN}\;+\;\mathrm{FP}\right)}}$$

where TP, FP, TN, and FN represent the true positives, false positives, false negatives and true negatives, respectively. Additionally, we plotted the ROC curves and calculated AUC to evaluate the performance of the predictors. The AUC with a < 10% FP rate (AUC01) was also calculated to reflect the prediction performance when the FP rate is low, which is more practical for experimental verification.

### Statistical methods

Student’s t-test was used to compare the means of two populations and ANOVA was used for the comparison for more than two populations. As for multiple comparisons, adjusted *P* value with the Benjamini–Hochberg (BH) method was adopted.

## Results and discussion

### The EAAC encoding performed the best among the encoding schemes examined

**Figure 1:**
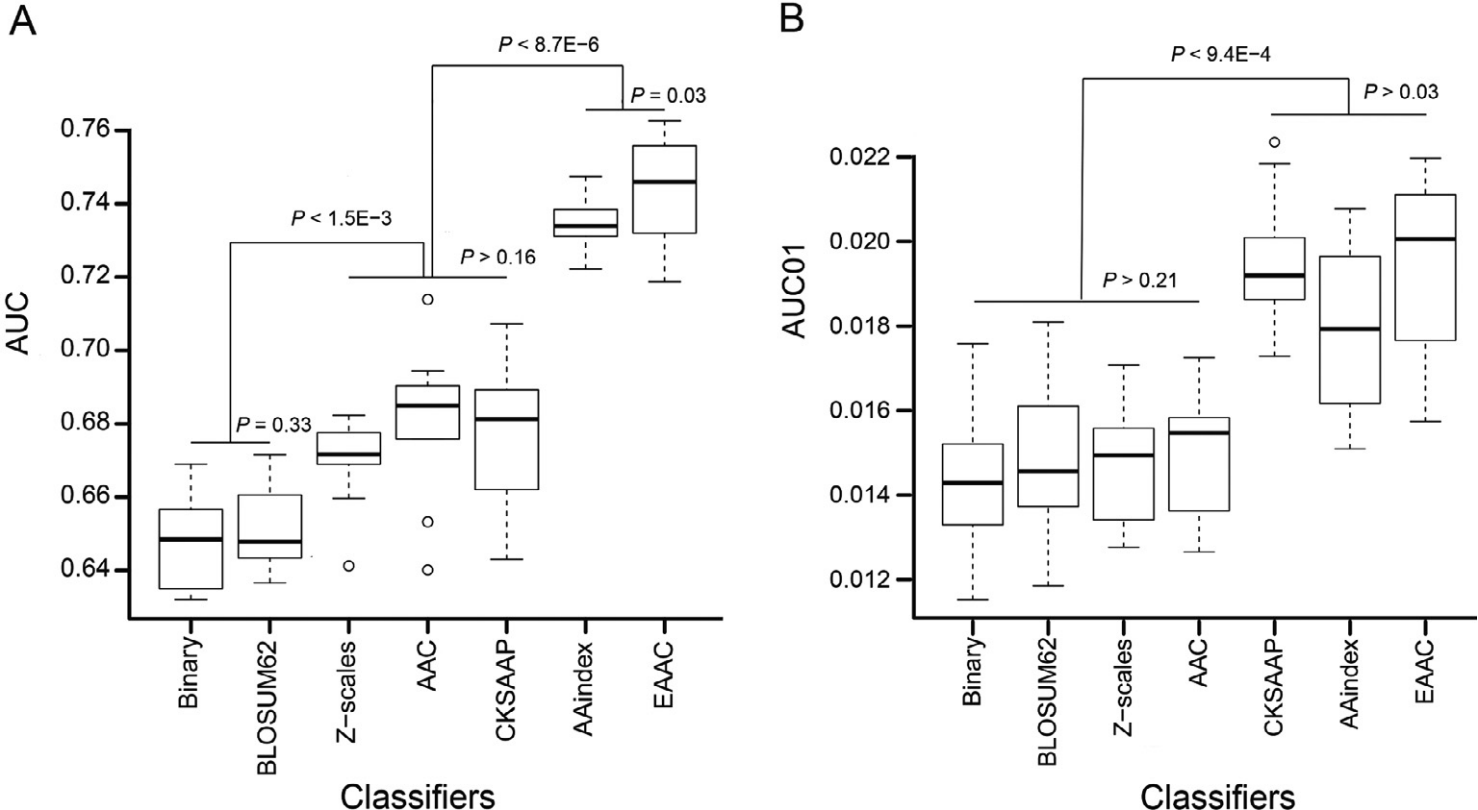
**Performance comparison of the Kmal predictors** The performance of different RF-based Kmal predictors were compared in terms of AUC (**A**) and AUC01 (**B**), respectively, for ten-fold cross-validation. *P* values were calculated using a paired Student’s *t*-test. AUC, area under the receiver operating characteristic; AUC01, AUC at a false positive rate below 10% (*i.e.*, specificity > 90%). A detailed performance comparison using different measurements is provided in Table S3.

Many computational approaches have been developed for the prediction of PTM sites. They are generally based on different ML algorithms combined with various pre-defined features encoded from peptide sequences. We reason that although the accuracy of a prediction approach is affected by the selection of the ML method, the major determinant likely comes from the encoding scheme. Accordingly, we constructed RF-based predictors with different common encoding schemes to evaluate these encodings for the Kmal prediction. The encoding schemes tested include BLOSUM62 [32], CKSAAP [33], [34], Binary [35], Z-scales [36], AAindex [18], AAC [27], and EAAC that was newly developed in this study. Among these different encoding schemes, the EAAC encoding performed the best in the prediction of Kmal sites for ten-fold cross-validation and the independent test, in terms of AUC, Ac, Sn, Sp, and MCC (Figure 1A and Table S3). As prediction performance at a low false positive rate is highly useful in practice, we estimated these predictors using AUC01, where the specificity was determined to be >90%. EAAC again showed the best performance for both ten-fold cross-validation and the independent test (Figure 1B and Table S3).

**Figure 2:**
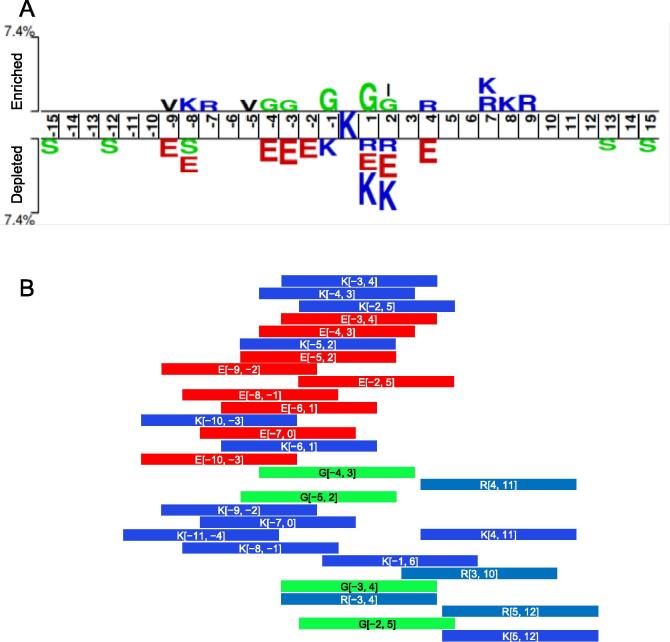
**Informative features in EAAC encoding** **A.** Sequence pattern surrounding the Kmal sites, including the significantly enriched and depleted residues based on Kmal peptides and non-modification peptides (*P* < 0.05, t-test with Bonferroni correction). The pattern was generated using the two-sample-logo method [37]. **B.** The informative features in EAAC encoding were ranked using the information gain method, with the top 30 features listed.

To explore the informative features in the EAAC encoding, we investigated the enrichment of residues at specific positions. We calculated the statistical significance of the position-specific residue frequencies between the positive (5288) and negative (88,636) peptides (Figure S1) [37]. Figure 2A shows the significantly enriched or depleted residues ranging from position −15 to +15. Similar to the previous Kmal analysis [10], the polar amino acid glycine (G) was generally enriched from position −4 to +2 in positive peptides, and basic lysine (K) was depleted from position −1 to +2. Different from the aforementioned study [10], we observed a significant enrichment of glutamic acid (E) from positions −4 to +4 except at positions −1 and +3 in negative peptides.

To further explore the most informative features, we ranked the sequence features using the information gain method [33], [38], [39] and selected top 30 features (Figure 2B). Interestingly, these features only involved four types of residues: three charged residues, (*i.e.*, K, R, and E) and the neutral residue (G). K and E were found in 13 and 9 features, respectively, and the remaining 8 features were equally divided by R and G. We compared these features with the sequence pattern surrounding the Kmal sites (Figure 2A) and found that the data are consistent. For instance, G was significantly enriched in the sequence positions −4 to +2. Similarly, this position range was covered by the top features ‘G[−5, 2]’, ‘G[−4, 3]’, ‘G[−3, 4]’, and ‘G[−2, 5]’. Additionally, K was depleted in the sequence positions from −1 to +2 and consistently enclosed by the features ‘K[−5, 2]’, ‘K[−4, 3]’, ‘K[−3, 4]’, and ‘K[−2, 5]’. Therefore, the performance of an EAAC-based RF classifier likely depends primarily on the extent to which the EAAC encoding accurately characterizes the flanking residues around Kmal sites.

### The DL approach with word embedding showed superior performance

**Figure 3:**
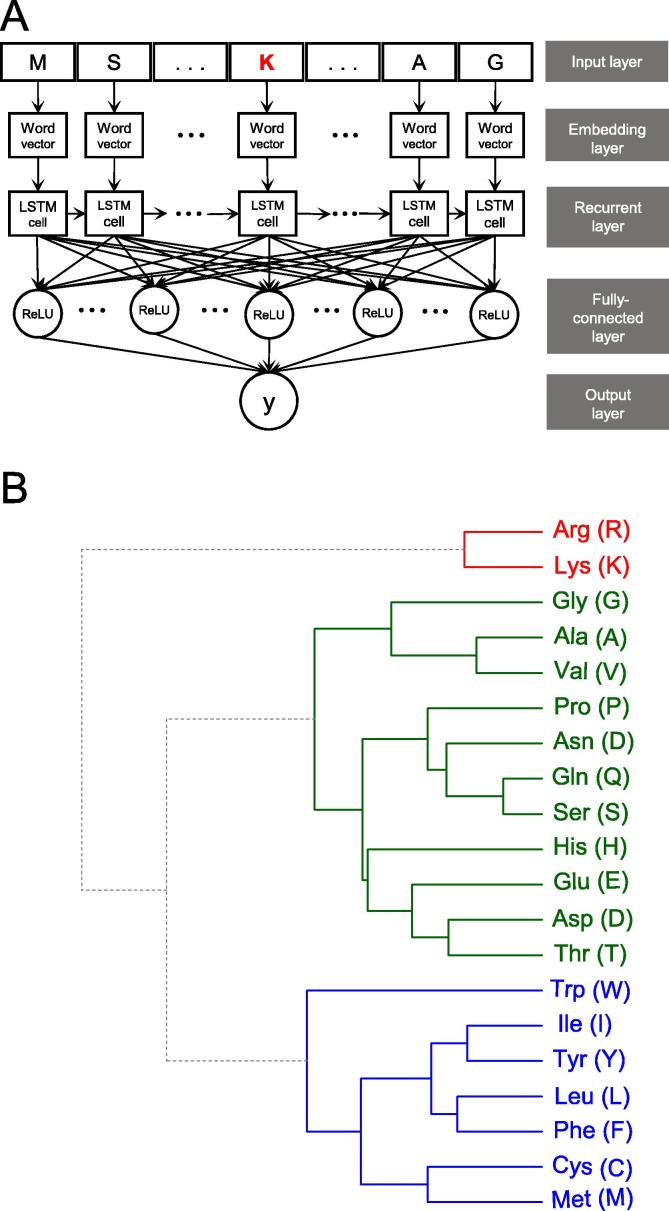
**The architecture of LSTM_WE_ and classification of the amino acids based on the information from LSTM_WE_** **A.** The LSTM-based DL classifier LSTM_WE_ contained five layers. The input layer received a peptide sequence of 31 residues with K in the center. In the embedding layer, each residue of the sequence was converted into a five-dimension word vector. In the recurrent layer, each of the 31 word vectors was input sequentially into the LSTM cell that contained 32 hidden neuron units. In the fully connected layer, 128 neuron units were built in which the ReLU was chosen for its activation function. The last layer included a single unit that output the probability “y” of Kmal modification. **B.** Hierarchical clustering of the 20 residues based on their related five-dimensional word vectors in the embedding layer and the calculation of Euclidean distance in average linkage. The residues were grouped into three major groups: (i) the alkaline residues K and R (red color), (ii) the aromatic and larger hydrophobic residues (blue color), and (iii) the remaining residues, including all acidic residues (green color).

The general PTM prediction approaches are based on traditional ML algorithms where pre-defined features are determined. In recent years, DL algorithms have been developed and applied to the field of PTM prediction [17], [18]. Here, we developed a DL classifier based on LSTM with the word embedding approach [40], named as LSTM_WE_, for the prediction of Kmal sites. This classifier contained five layers (Figure 3A). These include (1) input layer, in which the 31 residues of the peptide sequence fragments were considered categorical features; (2) embedding layer, in which each amino acid residue (including the gap ‘-’) was converted into a five-dimension word vector to represent amino acid properties, since word vectors have been utilized in a natural language process by embedment into neural networks [41]; (3) recurrent layer, in which each of the 31 word vectors was input sequentially into the LSTM cell that contained 32 hidden neuron units; (4) fully connected layer, in which 128 neuron units were built with the rectified linear unit (ReLU) chosen for its activation function; and (5) output layer, in which a single unit is activated by the “sigmoid” function, outputting the probability score. A peptide was predicted as positive if the probability score was larger than a specified threshold (*e.g.*, the threshold is 0.152 with Sp as 90%).

The parameters in the LSTM_WE_ network was trained and optimized based on binary cross-entropy loss function using the Adam algorithm [42]. The maximum of the training cycles was set as 300 epochs to ensure that the loss function value converged. In each epoch, the training dataset was separated with the batch size as 512 and iterated. To avoid overfitting, the dropout [43] rate of the neuron units was set as 20% after the recurrent and fully connected layers, respectively. The entire model was implemented by Tensorflow [44].

Recurrent neural networks are widely applied to the natural language process where every word is generally converted into a low-dimension vector instead of a one-hot vector to dissect the connotation of contexts [41]. This method avoids having a sparse vector space and readily infers the semantic similarity of words. In this study, we applied this concept to peptide sequences. Each amino acid was converted into a five-dimension word vector in the embedding layer. Finally, a 21 × 5 matrix was generated after training where every row represented a five-dimensional word vector of the amino acid. To investigate the similarity of amino acid residues around the Kmal sites, the 20 amino acids were hierarchically clustered using Euclidean distance in average linkage. Figure 3B shows that the amino acids were distributed into three clusters: (i) the alkaline residues K and R, (ii) the aromatic and large hydrophobic residues, and (iii) the remaining residues, including all acidic residues. The separation of acidic and alkaline residues indicated that the acid-base property of residues played a key role in influencing Kmal, which was in line with the observation that the distribution of K and R was significantly lopsided (Figure 2A). Moreover, all the aromatic residues and some hydrophobic residues were aggregated into one cluster, while some residues with a smaller side chain volume, such as A, G and V, formed a subclass in another cluster. This implies that the size of a residue might also affect Kmal. All the results demonstrate that our model is capable of elucidating the significance of the correlation between amino acid properties and Kmal.

**Figure 4:**
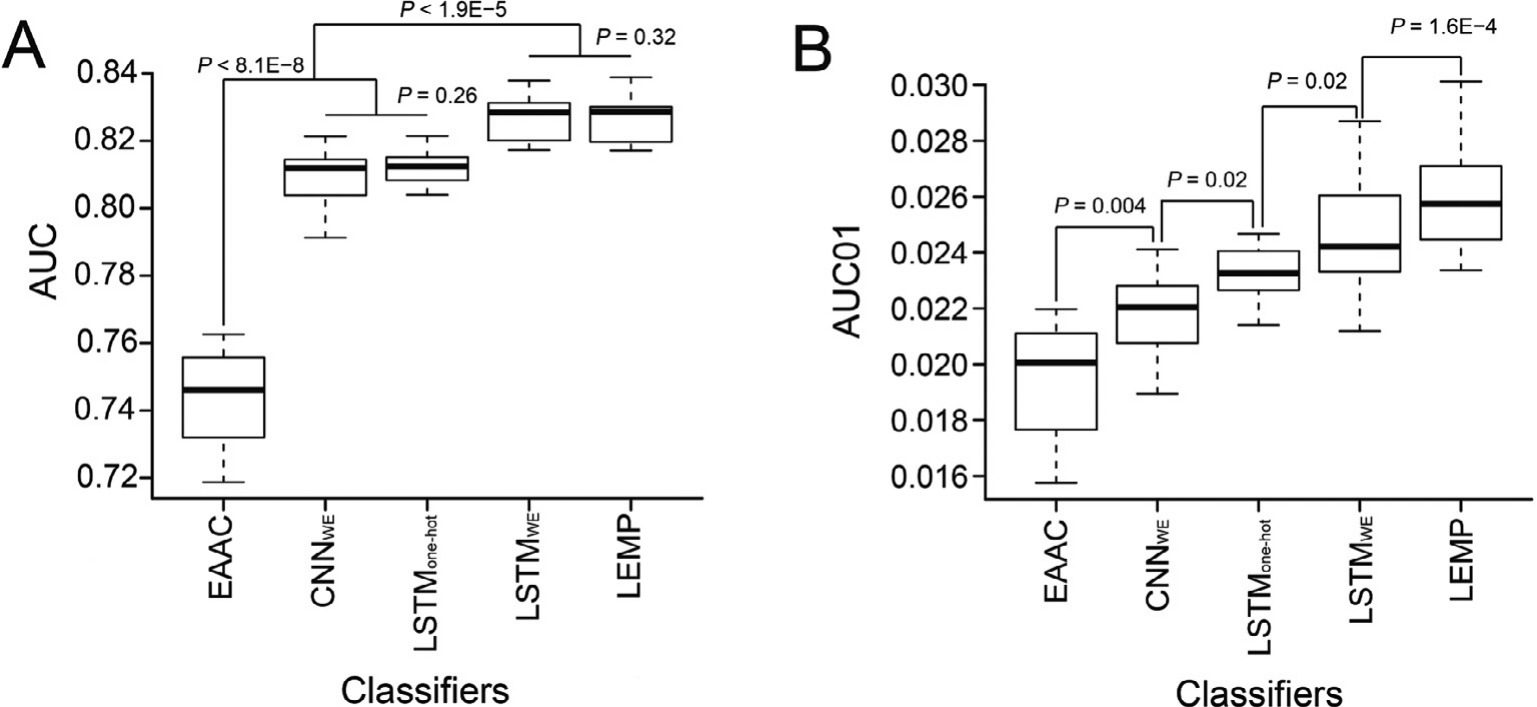
**Performance comparison of the DL-based Kmal predictors** The performance of different DL-based Kmal predictors were compared in terms of AUC (**A**) and AUC01 (**B**), respectively, for ten-fold cross-validation. *P* values were calculated using a paired student’s t-test. A detailed performance comparison using different measurements is provided in Table S3.

Compared to the traditional classifier RF_EAAC_ described earlier, the LSTM_WE_ method had the largest AUC, AUC01, Ac, Sn, Sp, and MCC values for both the ten-fold cross-validation and the independent test (Figure 4 and Table S3), suggesting that the DL classifier precisely captured the unique information from Kmal-containing peptides. LSTM is a self-taught representation learning algorithm in that it not only employs the local sequence pattern via short term memory but, more importantly, also extracts effective information from the non-local residue correlation via long term memory. This may explain why LSTM demonstrated superior performance.

To compare the performance of LSTM_WE_ with other DL frameworks, we developed the convolution neural network (CNN)-based classifier named CNN_WE_, which included an embedding layer as input, four convolution layers as the hidden layer, and an output layer. The same parameter optimization strategy used in LSTM_WE_ was adopted for CNN_WE_. LSTM_WE_ performed better than CNN_WE_ (Figure 4 and Table S3). Moreover, we developed the LSTM-based DL classifier with one-hot encoding, dubbed LSTM_one-hot_, where the word embedding layer in LSTM_WE_ was replaced by one-hot encoding. LSTM_WE_ compared favorably to LSTM_one-hot_ in terms of AUC and AUC01 values (Figure 4 and Table S3).

### Establishment of the LEMP by integrating LSTMWE and RFEAAC

**Figure 5:**
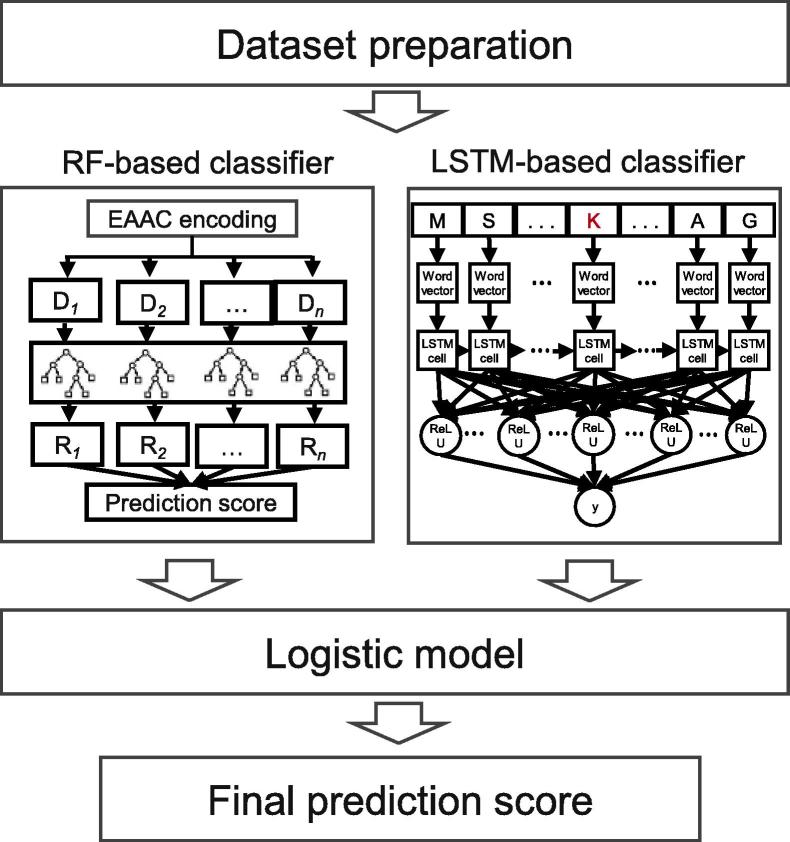
**The framework of LEMP** LEMP was established by integrating LSTM_WE_ and RF_EAAC_ using the logistical regression approach (see methods for details). In RF_EAAC_, D*_n_* represents the *n*-th decision tree and R*_n_* represents the result of *n*th decision tree. The input dataset was pre-processed to extract 31 amino acid sequences with the Ks to be predicted in the center. Each sequence was then read by the integrated LEMP and then the K in the center had the prediction score.

We showed above that LSTM_WE_ outperformed various classifiers with different feature encodings. Due to the potential complementary effects in combining different classifiers to achieve better results, we investigated whether an integration of two classifiers would be more robust or perform better. We developed LEMP by integrating LSTM_WE_ and the EAAC-encoding RF classifier using the logistical regression approach (Figure 5). LEMP showed outstanding performance for both cross-validation and the independent test in terms of AUC01, MCC, and Sn values, although they had similar values of AUC, Ac, and Sp (Figure 4, Table S3, and Figure S4). Additionally, we developed LEMP separately for humans and mice. We found that the individual LEMP models performed similarly to the integrated models (*P* > 0.05; data not shown). Therefore, we integrated both species in this study.

### Estimation of the impact of data size on prediction accuracy

**Figure 6:**
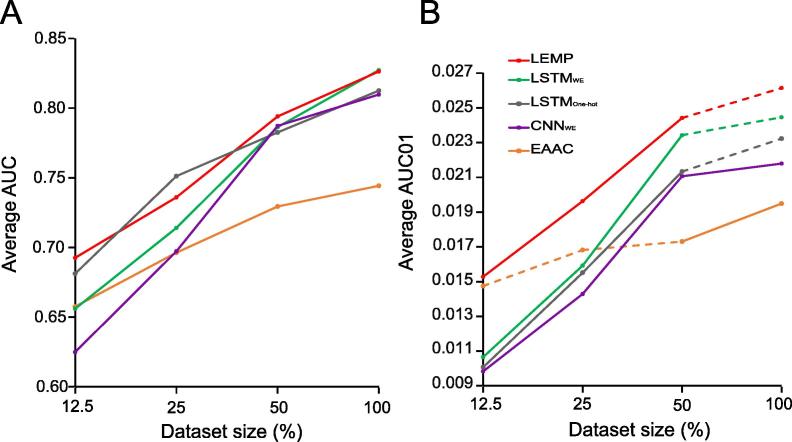
**Estimation of the impact of data size on prediction accuracy** The average AUC values (**A**) and average AUC01 values (**B**) were calculated using four different data sizes: an eighth, a quarter, a half, and the whole dataset (containing 4242 positive peptides and 71,809 negative peptides; Figure S1) for ten-fold cross-validation. For each algorithm, the AUC or AUC01 values between the adjacent datasets were statistically compared. The solid line represents significant differences (*P* < 0.01, *P* value with BH adjustment), and the dashed line represents non-significant differences.

The performance of a ML algorithm is generally sensitive to the size of the training data. To compare the sensitivities of the algorithms described above, we calculated their performances constructed based on an eighth (9506), a quarter (19,012), a half (38,025) of, and the whole (76,051) training dataset with ten-fold cross-validation (Figure S1), separately Figure 6 shows that although the overall performances of all the approaches increased with the size of the training dataset, the DL algorithms (*i.e.*, LSTM_One-hot_ and LSTM_WE_) performed better than the traditional algorithm RF_EAAC_ in terms of AUC and AUC01 values. The DL algorithms had larger AUC01 values than RF_EAAC_ for large-sized dataset but not for small-sized dataset (Figure 6B). The performance of LEMP was similar to that of LSTM_WE_ when using the whole training dataset, but the former compared favorably to the latter with the small size of the training dataset (Figure 6A). This result indicates that LSTM_WE_ built using the small data size has a relatively low performance but that the performance could be improved by integrating RF_EAAC_. With an increased data size, the contribution of RF_EAAC_ to the prediction performance decreases, while that of LSTM_WE_ increases. A similar observation was made for the comparison using the AUC01 values (Figure 6B). These results suggest that DL algorithms built with the small training set performs relatively better than the traditional ML methods, and their performance is improved by integrating traditional methods, because as the dataset increases in size, the accuracy of the DL algorithms increases at a faster rate. As the performance of LEMP is significantly better than LSTM_WE_ in terms of AUC01 (Figure 4B), we selected LEMP for our following study.

### Comparison of LEMP with reported Kmal predictors

**Figure 7:**
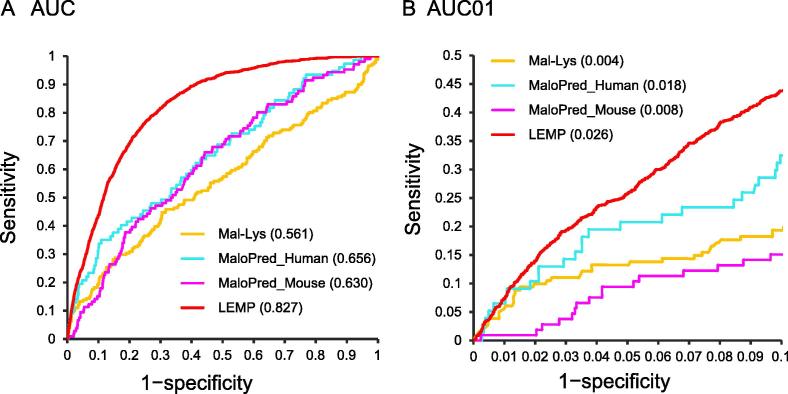
**Performance of comparison of LEMP with Mal-Lys and MaloPred** AUC (**A**) and AUC01 (**B**) curves were generated for the predictors using the independent test dataset. The values for AUC and AUC01 obtained using different methods were indicated in the parenthesis, respectively.

We assessed the performance of LEMP by comparing it with the currently available Kmal predictors, Mal-Lys [10] and MaloPred [11], based on our independent test dataset (see Methods for details). MaloPred contained two different prediction algorithms, one for humans (*i.e.*, MaloPred_Human) and the other for mice (*i.e.*, MaloPred_Mouse). We adjusted the test dataset (final positive peptides: 183; negative peptides: 4004) by removing the sequences that were used for training the published algorithms. As a result, LEMP outperformed the competitors in terms of AUC and AUC01 values (Figure 7). The independent dataset (containing 1046 positive peptides and 16,827 negative peptides) was used for comparison with Mal-Lys. As a result, LEMP achieved an AUC of 0.827 (AUC01 = 0.026), while the AUC value of Mal-Lys is 0.561 (AUC01 = 0.004).

## Conclusions

The currently available PTM prediction approaches are mainly based on ML that requires experts to pre-define informative features. Here, we applied the DL methodology to PTM prediction and developed an LSTM-based classifier for predicting malonylation sites. Despite lacking pre-defined features, the DL classifier demonstrated a superior performance compared to the traditional ML methods. This was likely due to the strong capability of the DL methodology to learn sparse representation in a self-taught manner; thus, the DL classifier could auto-capture the most informative features. The DL methodology is sensitive to the homogeneity and size of samples, but this limitation can be overcome by integration with a traditional ML classifier. The outstanding performance of DL in the prediction of Kmal sites suggests that DL may be applied broadly to predicting other types of PTM sites.
