# Supplementary material for: Integration of A Deep Learning Classifier with A Random Forest Approach for Predicting Malonylation Sites
Source: Genomics Proteomics Bioinformatics. 2019 Jan 11;16(6):451–9. doi: 10.1016/j.gpb.2018.08.004 (PMC6411950; doi:10.1016/j.gpb.2018.08.004)
Supplement: Supplementary data 1 [file mmc1.pptx]

## Slide 1
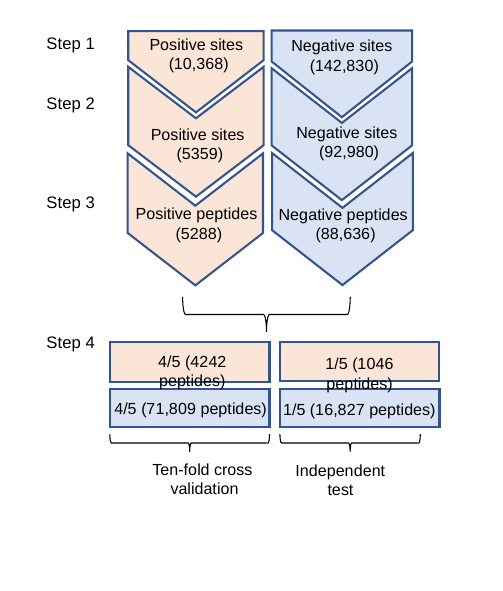

Step 1
Step 2
Step 3
Step 4
Positive sites
 (10,368)
Negative sites
 (142,830)
Negative sites
 (92,980)
Positive sites
 (5359)
Positive peptides
 (5288)
Negative peptides
 (88,636)
4/5 (4242 peptides)
1/5 (1046 peptides)
4/5 (71,809 peptides)
1/5 (16,827 peptides)
Ten-fold cross
validation
Independent
test
