# Supplementary figures and images for: Integration of A Deep Learning Classifier with A Random Forest Approach for Predicting Malonylation Sites
Source: Genomics Proteomics Bioinformatics. 2019 Jan 11;16(6):451–9. doi: 10.1016/j.gpb.2018.08.004 (PMC6411950; doi:10.1016/j.gpb.2018.08.004)

## Slide 1
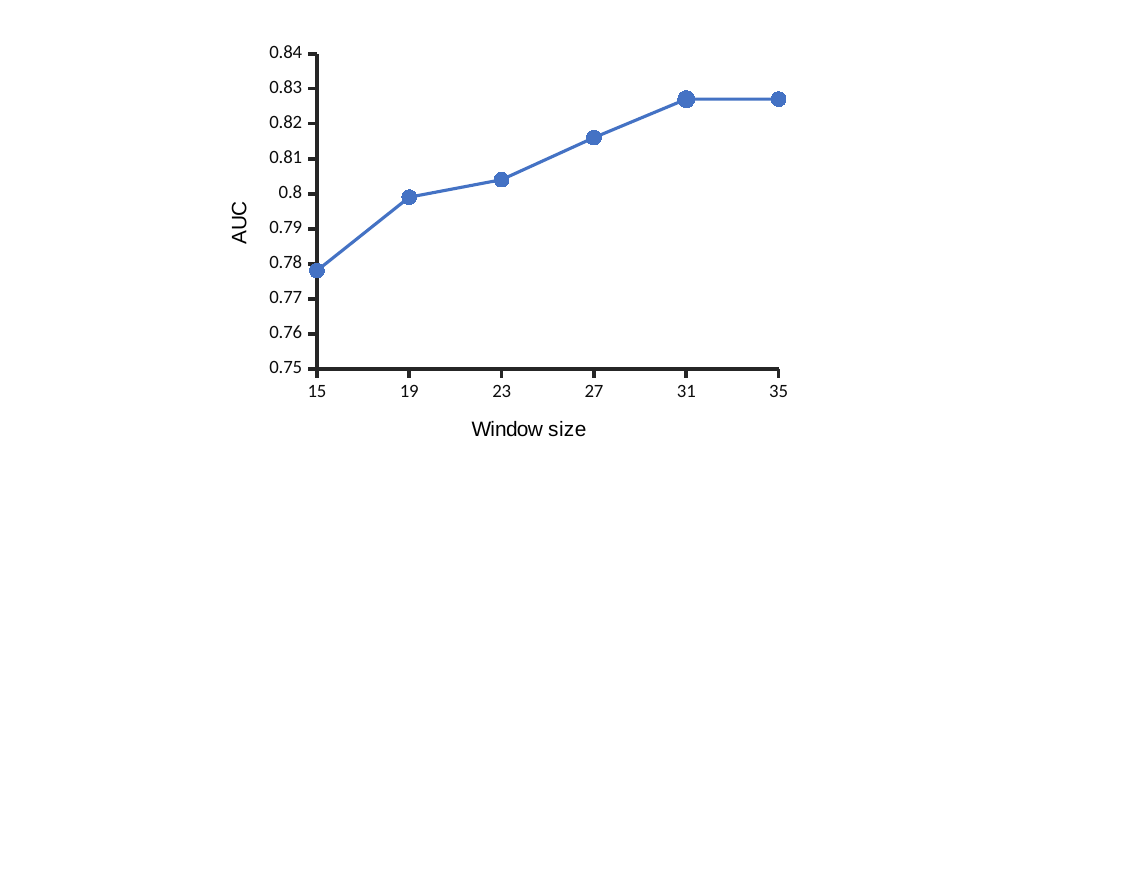

### Chart
| Category | |
|---|---|

Supplement: Supplementary data 2 [file mmc2.pptx]

## Slide 1
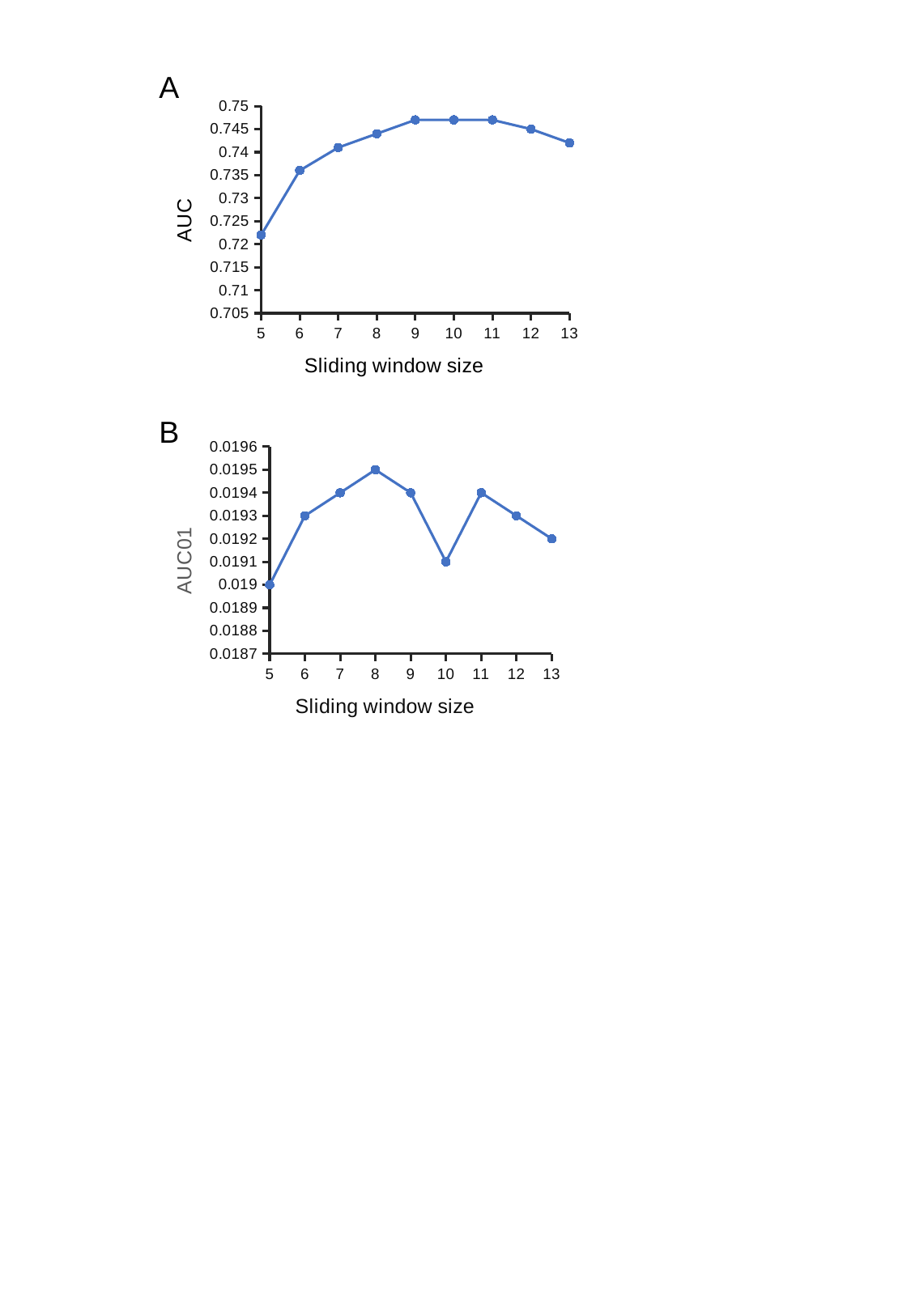

A
### Chart
| Category | |
|---|---|B
### Chart
| Category | |
|---|---|

Supplement: Supplementary data 3 [file mmc3.pptx]
