# Supplementary material for: Integration of A Deep Learning Classifier with A Random Forest Approach for Predicting Malonylation Sites
Source: Genomics Proteomics Bioinformatics. 2019 Jan 11;16(6):451–9. doi: 10.1016/j.gpb.2018.08.004 (PMC6411950; doi:10.1016/j.gpb.2018.08.004)
Supplement: Supplementary data 4 [file mmc4.pdf]

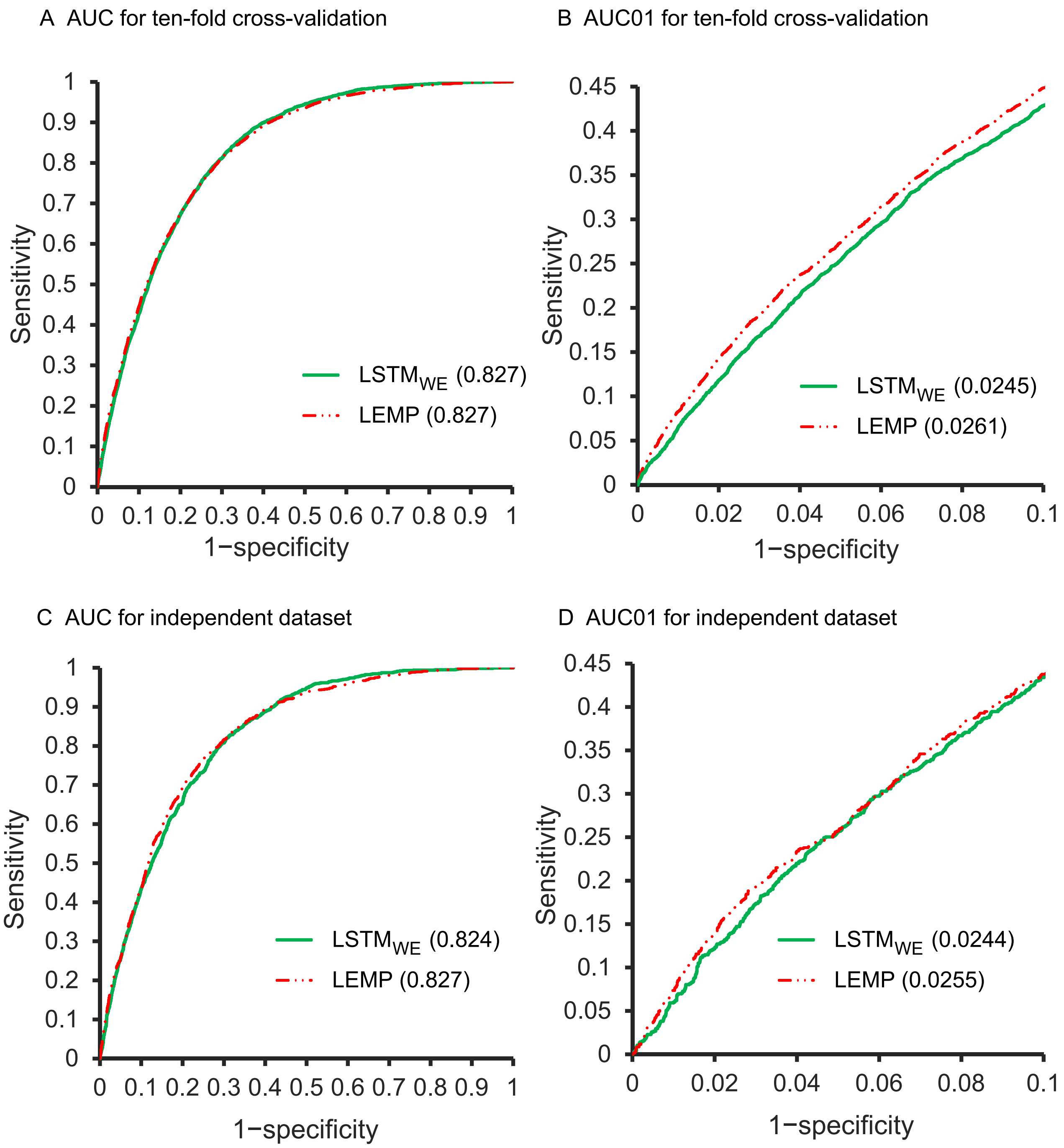

A AUC for ten-fold cross-validation
B AUC01 for ten-fold cross-validation
C AUC for independent dataset
D AUC01 for independent dataset
Sensitivity
1−specificity
LSTMWE (0.827)
LEMP (0.827)
LSTMWE (0.0245)
LEMP (0.0261)
LSTMWE (0.824)
LEMP (0.827)
LSTMWE (0.0244)
LEMP (0.0255)
