# Supplementary material for: Integration of A Deep Learning Classifier with A Random Forest Approach for Predicting Malonylation Sites
Source: Genomics Proteomics Bioinformatics. 2019 Jan 11;16(6):451–9. doi: 10.1016/j.gpb.2018.08.004 (PMC6411950; doi:10.1016/j.gpb.2018.08.004)
Supplement: Supplementary data 6 [file mmc6.docx]

**Table S2 AUC values for each of the 531 AAindex properties using the RF classifier**

| **Serial No.** | **Physiochemical property** | **AUC** |
| --- | --- | --- |
|  | RICJ880113 | 0.706145 |
|  | CHOC760103 | 0.703819 |
|  | JANJ780102 | 0.703297 |
|  | NADH010102 | 0.703045 |
|  | LEVM760101 | 0.702804 |
|  | ROSM880102 | 0.702285 |
|  | FINA910104 | 0.70197 |
|  | JANJ790101 | 0.701724 |
|  | CHOC760102 | 0.701615 |
|  | NADH010103 | 0.701524 |
|  | KIDA850101 | 0.700525 |
|  | FINA910102 | 0.699799 |
|  | GARJ730101 | 0.699667 |
|  | HUTJ700103 | 0.699539 |
|  | FAUJ830101 | 0.69935 |
|  | GUYH850101 | 0.699036 |
|  | OOBM770101 | 0.698704 |
|  | JANJ790102 | 0.698365 |
|  | CHOC760104 | 0.698225 |
|  | GUYH850104 | 0.698112 |
|  | JANJ780103 | 0.698006 |
|  | KYTJ820101 | 0.697231 |
|  | FINA910103 | 0.69711 |
|  | GUYH850105 | 0.696751 |
|  | OLSK800101 | 0.696149 |
|  | EISD860102 | 0.695532 |
|  | JURD980101 | 0.695434 |
|  | JANJ780101 | 0.69536 |
|  | JACR890101 | 0.695184 |
|  | NAKH900110 | 0.694538 |
|  | RADA880108 | 0.693827 |
|  | RADA880107 | 0.6935 |
|  | EISD840101 | 0.692398 |
|  | ZIMJ680104 | 0.685936 |
|  | FAUJ880104 | 0.684871 |
|  | KLEP840101 | 0.6821 |
|  | NAKH920108 | 0.679791 |
|  | WOLS870103 | 0.678808 |
|  | WILM950103 | 0.678643 |
|  | HUTJ700102 | 0.677049 |
|  | FASG760103 | 0.676719 |
|  | COWR900101 | 0.675233 |
|  | WILM950101 | 0.671541 |
|  | FASG890101 | 0.663078 |
|  | NADH010104 | 0.66043 |
|  | RACS770102 | 0.658414 |
|  | MITS020101 | 0.65652 |
|  | AURR980116 | 0.655148 |
|  | KRIW710101 | 0.65394 |
|  | KRIW790101 | 0.652971 |
|  | RADA880105 | 0.652568 |
|  | GUOD860101 | 0.652355 |
|  | DAWD720101 | 0.651455 |
|  | LEVM760105 | 0.651221 |
|  | NAKH900111 | 0.648988 |
|  | ENGD860101 | 0.648887 |
|  | BASU050103 | 0.646997 |
|  | PRAM900101 | 0.646932 |
|  | MIYS990105 | 0.646861 |
|  | NAKH920105 | 0.646089 |
|  | OOBM850103 | 0.645606 |
|  | ZIMJ680103 | 0.645498 |
|  | MEIH800102 | 0.64485 |
|  | OOBM770102 | 0.644769 |
|  | PUNT030101 | 0.644429 |
|  | FUKS010104 | 0.644066 |
|  | PONP800101 | 0.640902 |
|  | MIYS990104 | 0.639596 |
|  | CORJ870101 | 0.639531 |
|  | ROSG850102 | 0.639321 |
|  | WARP780101 | 0.63913 |
|  | MIYS990102 | 0.639112 |
|  | PONP800102 | 0.639066 |
|  | VHEG790101 | 0.638956 |
|  | PONP800103 | 0.638854 |
|  | MIYS990103 | 0.6385 |
|  | KRIW790102 | 0.638355 |
|  | ZHOH040103 | 0.638177 |
|  | FUKS010101 | 0.637577 |
|  | MIYS990101 | 0.637575 |
|  | PONP800108 | 0.637539 |
|  | BIOV880101 | 0.637146 |
|  | PONP930101 | 0.637043 |
|  | FAUJ880109 | 0.636611 |
|  | ZIMJ680105 | 0.636234 |
|  | LEVM760102 | 0.636157 |
|  | MEIH800103 | 0.635607 |
|  | RACS820103 | 0.635482 |
|  | VINM940104 | 0.635468 |
|  | PRAM820102 | 0.635371 |
|  | DESM900102 | 0.635338 |
|  | VINM940101 | 0.635148 |
|  | FUKS010108 | 0.634873 |
|  | NAKH900112 | 0.634866 |
|  | MIYS850101 | 0.634687 |
|  | RADA880103 | 0.634169 |
|  | PONP800107 | 0.63404 |
|  | BIOV880102 | 0.633865 |
|  | DESM900101 | 0.63356 |
|  | CASG920101 | 0.632782 |
|  | NISK860101 | 0.631948 |
|  | AURR980117 | 0.631821 |
|  | WERD780101 | 0.631549 |
|  | LEVM760103 | 0.63133 |
|  | BLAS910101 | 0.631094 |
|  | RADA880104 | 0.630842 |
|  | RADA880101 | 0.630712 |
|  | OOBM850105 | 0.630532 |
|  | RACS770103 | 0.630023 |
|  | CHOC760101 | 0.629773 |
|  | HOPA770101 | 0.629672 |
|  | NAKH900113 | 0.629635 |
|  | HOPT810101 | 0.629386 |
|  | WOLR810101 | 0.6291 |
|  | WOEC730101 | 0.628616 |
|  | HARY940101 | 0.628192 |
|  | WOLR790101 | 0.628035 |
|  | CHOP780205 | 0.62796 |
|  | PONP800106 | 0.62707 |
|  | PRAM820101 | 0.62685 |
|  | CHOC750101 | 0.626795 |
|  | GRAR740103 | 0.626772 |
|  | ARGP820102 | 0.626461 |
|  | EISD860101 | 0.6262 |
|  | GOLD730102 | 0.625962 |
|  | PONP800105 | 0.625835 |
|  | KRIW790103 | 0.625328 |
|  | CORJ870106 | 0.62532 |
|  | MEIH800101 | 0.625019 |
|  | FAUJ880111 | 0.624938 |
|  | OOBM770103 | 0.624818 |
|  | ROSM880101 | 0.624707 |
|  | WEBA780101 | 0.624665 |
|  | RADA880106 | 0.624379 |
|  | GEOR030105 | 0.624043 |
|  | FAUJ880106 | 0.624011 |
|  | LEVM760107 | 0.624004 |
|  | YUTK870101 | 0.62375 |
|  | FUKS010107 | 0.623639 |
|  | TSAJ990102 | 0.62296 |
|  | KUHL950101 | 0.622651 |
|  | BIGC670101 | 0.622585 |
|  | NAKH900106 | 0.622255 |
|  | WOLS870102 | 0.622239 |
|  | PUNT030102 | 0.622041 |
|  | WERD780103 | 0.621812 |
|  | GUYH850102 | 0.621809 |
|  | FAUJ880103 | 0.621805 |
|  | NAKH900104 | 0.621478 |
|  | GRAR740102 | 0.621177 |
|  | WOLS870101 | 0.621086 |
|  | CHAM830106 | 0.621006 |
|  | FASG760101 | 0.619587 |
|  | TANS770106 | 0.619268 |
|  | NAKH900105 | 0.618972 |
|  | KANM800102 | 0.618886 |
|  | ONEK900102 | 0.618839 |
|  | FINA910101 | 0.618106 |
|  | TSAJ990101 | 0.617903 |
|  | HUTJ700101 | 0.617835 |
|  | ARGP820103 | 0.617724 |
|  | FUKS010102 | 0.617711 |
|  | BAEK050101 | 0.617615 |
|  | TAKK010101 | 0.617586 |
|  | PALJ810104 | 0.617414 |
|  | VINM940103 | 0.617288 |
|  | NADH010101 | 0.616769 |
|  | PTIO830102 | 0.616756 |
|  | TANS770107 | 0.61649 |
|  | NAKH900103 | 0.615583 |
|  | RACS820105 | 0.615567 |
|  | RICJ880115 | 0.615037 |
|  | SNEP660103 | 0.6142 |
|  | EISD860103 | 0.613959 |
|  | CHAM820101 | 0.613929 |
|  | PALJ810105 | 0.613376 |
|  | RADA880102 | 0.613186 |
|  | RACS820106 | 0.613091 |
|  | KARP850103 | 0.61299 |
|  | MEEJ810102 | 0.612913 |
|  | FAUJ880101 | 0.612331 |
|  | YUTK870104 | 0.612299 |
|  | TANS770109 | 0.612076 |
|  | FUKS010103 | 0.611994 |
|  | ZHOH040102 | 0.611932 |
|  | WIMW960101 | 0.611183 |
|  | MCMT640101 | 0.611051 |
|  | PLIV810101 | 0.61076 |
|  | PALJ810106 | 0.610519 |
|  | CORJ870102 | 0.610169 |
|  | YUTK870102 | 0.610006 |
|  | ISOY800103 | 0.609968 |
|  | JOND750102 | 0.609856 |
|  | MAXF760102 | 0.609807 |
|  | MEEJ800102 | 0.609747 |
|  | QIAN880113 | 0.60958 |
|  | FASG760102 | 0.609408 |
|  | BASU050102 | 0.609254 |
|  | ISOY800102 | 0.609247 |
|  | BHAR880101 | 0.609026 |
|  | QIAN880129 | 0.608708 |
|  | NISK800101 | 0.608646 |
|  | ZHOH040101 | 0.608553 |
|  | CHOP780207 | 0.608369 |
|  | KIMC930101 | 0.608296 |
|  | RICJ880112 | 0.608109 |
|  | FUKS010106 | 0.608017 |
|  | GEIM800110 | 0.607947 |
|  | DIGM050101 | 0.60767 |
|  | RACS820101 | 0.607658 |
|  | SNEP660104 | 0.607129 |
|  | ZIMJ680102 | 0.607121 |
|  | VINM940102 | 0.607016 |
|  | CIDH920104 | 0.606921 |
|  | YUTK870103 | 0.606921 |
|  | MANP780101 | 0.606692 |
|  | CHOP780202 | 0.606379 |
|  | KOEP990101 | 0.606356 |
|  | LEVM760106 | 0.606188 |
|  | RICJ880108 | 0.606079 |
|  | OOBM770105 | 0.606062 |
|  | NAGK730102 | 0.605965 |
|  | LEVM780105 | 0.605874 |
|  | AURR980120 | 0.605771 |
|  | AURR980119 | 0.605541 |
|  | LEVM760104 | 0.605328 |
|  | PARS000101 | 0.605243 |
|  | BROC820101 | 0.605233 |
|  | GEIM800107 | 0.605115 |
|  | CORJ870107 | 0.605045 |
|  | SNEP660101 | 0.605013 |
|  | CHOP780204 | 0.604929 |
|  | ROBB760102 | 0.604545 |
|  | QIAN880122 | 0.604533 |
|  | ONEK900101 | 0.604509 |
|  | KUMS000101 | 0.604471 |
|  | BLAM930101 | 0.604435 |
|  | QIAN880134 | 0.604372 |
|  | CIDH920105 | 0.604249 |
|  | SWER830101 | 0.604169 |
|  | NAKH920106 | 0.60411 |
|  | PONJ960101 | 0.604075 |
|  | CIDH920102 | 0.603876 |
|  | CHOP780206 | 0.603805 |
|  | CHOP780203 | 0.603792 |
|  | FAUJ880102 | 0.60361 |
|  | FUKS010105 | 0.6036 |
|  | CORJ870108 | 0.60345 |
|  | CHOP780101 | 0.603446 |
|  | ISOY800105 | 0.603425 |
|  | CHAM830101 | 0.603299 |
|  | AURR980114 | 0.603241 |
|  | ROBB760113 | 0.603209 |
|  | ROBB760104 | 0.60317 |
|  | FASG760105 | 0.602997 |
|  | QIAN880112 | 0.60277 |
|  | LEVM780106 | 0.602674 |
|  | WERD780104 | 0.602629 |
|  | GEIM800105 | 0.602509 |
|  | PALJ810116 | 0.602478 |
|  | PRAM900104 | 0.602457 |
|  | ROBB760105 | 0.602323 |
|  | PALJ810110 | 0.602282 |
|  | CORJ870105 | 0.60213 |
|  | MONM990201 | 0.602042 |
|  | FAUJ880107 | 0.60184 |
|  | LEVM780103 | 0.601603 |
|  | PONP800104 | 0.601542 |
|  | VASM830103 | 0.601539 |
|  | QIAN880127 | 0.601478 |
|  | TANS770103 | 0.601354 |
|  | CHOP780211 | 0.601178 |
|  | BULH740101 | 0.600945 |
|  | RACS820113 | 0.600801 |
|  | GEIM800111 | 0.600488 |
|  | QIAN880101 | 0.60046 |
|  | FODM020101 | 0.600394 |
|  | KANM800104 | 0.600212 |
|  | LIFS790102 | 0.600053 |
|  | CHOP780216 | 0.600038 |
|  | RACS820114 | 0.599998 |
|  | AURR980104 | 0.599896 |
|  | CHOP780213 | 0.59986 |
|  | CHOP780209 | 0.599659 |
|  | BURA740102 | 0.59957 |
|  | CORJ870103 | 0.599548 |
|  | GEIM800106 | 0.599381 |
|  | AURR980102 | 0.599288 |
|  | ROBB760106 | 0.599155 |
|  | ROBB760110 | 0.598897 |
|  | BASU050101 | 0.598892 |
|  | QIAN880117 | 0.598828 |
|  | QIAN880131 | 0.59881 |
|  | PALJ810114 | 0.598685 |
|  | KUMS000102 | 0.598597 |
|  | GRAR740101 | 0.598554 |
|  | ZIMJ680101 | 0.598512 |
|  | ROBB760103 | 0.598431 |
|  | CORJ870104 | 0.59843 |
|  | AURR980113 | 0.598174 |
|  | WILM950102 | 0.598142 |
|  | RACS820102 | 0.598061 |
|  | MAXF760104 | 0.598033 |
|  | CHAM810101 | 0.597983 |
|  | QIAN880133 | 0.597978 |
|  | CHOP780208 | 0.597958 |
|  | LEVM780102 | 0.597719 |
|  | PRAM900103 | 0.597719 |
|  | SNEP660102 | 0.597683 |
|  | PALJ810112 | 0.59766 |
|  | NAKH900107 | 0.597568 |
|  | ZASB820101 | 0.597531 |
|  | MAXF760106 | 0.597526 |
|  | SIMZ760101 | 0.597299 |
|  | MUNV940103 | 0.597227 |
|  | CIDH920101 | 0.597203 |
|  | JOND750101 | 0.597169 |
|  | MEEJ810101 | 0.596949 |
|  | CHAM820102 | 0.596692 |
|  | PARJ860101 | 0.596645 |
|  | MONM990101 | 0.596557 |
|  | ISOY800104 | 0.59654 |
|  | ARGP820101 | 0.596305 |
|  | CHOP780210 | 0.596229 |
|  | LAWE840101 | 0.596175 |
|  | PARS000102 | 0.595999 |
|  | QIAN880107 | 0.59593 |
|  | ROBB760108 | 0.595825 |
|  | CHOP780215 | 0.595693 |
|  | OOBM770104 | 0.595532 |
|  | SUEM840101 | 0.595519 |
|  | NAKH900109 | 0.595476 |
|  | MEEJ800101 | 0.595471 |
|  | RACS770101 | 0.595365 |
|  | AURR980105 | 0.595349 |
|  | BULH740102 | 0.595276 |
|  | NAKH900108 | 0.595071 |
|  | GEIM800104 | 0.594935 |
|  | ROSG850101 | 0.594689 |
|  | RACS820104 | 0.594434 |
|  | KHAG800101 | 0.594431 |
|  | GEIM800101 | 0.593976 |
|  | RACS820111 | 0.593906 |
|  | RICJ880104 | 0.59383 |
|  | FUKS010111 | 0.593758 |
|  | AURR980106 | 0.593462 |
|  | FINA770101 | 0.592973 |
|  | ISOY800106 | 0.592883 |
|  | ISOY800107 | 0.592602 |
|  | NAKH900102 | 0.592574 |
|  | DAYM780201 | 0.592194 |
|  | CHOP780212 | 0.592187 |
|  | KOEP990102 | 0.59217 |
|  | ROSM880103 | 0.591803 |
|  | RICJ880105 | 0.591773 |
|  | MAXF760101 | 0.59157 |
|  | PALJ810109 | 0.591497 |
|  | WILM950104 | 0.59144 |
|  | CRAJ730102 | 0.591377 |
|  | PALJ810103 | 0.591045 |
|  | PRAM820103 | 0.590981 |
|  | ROBB760112 | 0.590981 |
|  | TANS770101 | 0.590955 |
|  | NADH010106 | 0.59094 |
|  | LIFS790103 | 0.590815 |
|  | GEIM800103 | 0.5908 |
|  | QIAN880132 | 0.590607 |
|  | CHOP780214 | 0.590593 |
|  | BEGF750101 | 0.590588 |
|  | RACS820110 | 0.590168 |
|  | QIAN880135 | 0.590167 |
|  | NADH010105 | 0.590094 |
|  | QIAN880115 | 0.590051 |
|  | PALJ810108 | 0.590013 |
|  | QIAN880121 | 0.589873 |
|  | CIDH920103 | 0.589741 |
|  | VASM830102 | 0.589552 |
|  | LIFS790101 | 0.589404 |
|  | SUYM030101 | 0.589359 |
|  | MAXF760105 | 0.589181 |
|  | PALJ810111 | 0.588967 |
|  | BROC820102 | 0.588775 |
|  | ANDN920101 | 0.588639 |
|  | NAKH920102 | 0.588434 |
|  | MAXF760103 | 0.588424 |
|  | AURR980110 | 0.588188 |
|  | GEOR030106 | 0.588119 |
|  | RICJ880109 | 0.588033 |
|  | MUNV940102 | 0.588032 |
|  | QIAN880139 | 0.587764 |
|  | CEDJ970105 | 0.587762 |
|  | MUNV940104 | 0.587757 |
|  | BURA740101 | 0.587754 |
|  | TANS770110 | 0.587507 |
|  | VASM830101 | 0.587497 |
|  | JOND920102 | 0.587464 |
|  | ISOY800108 | 0.587421 |
|  | AURR980111 | 0.587385 |
|  | KARP850101 | 0.58734 |
|  | AURR980118 | 0.587178 |
|  | COHE430101 | 0.587149 |
|  | QIAN880125 | 0.587065 |
|  | NAKH920107 | 0.586876 |
|  | RICJ880103 | 0.586232 |
|  | GEIM800108 | 0.586019 |
|  | CRAJ730103 | 0.585831 |
|  | ISOY800101 | 0.585767 |
|  | QIAN880106 | 0.585671 |
|  | BUNA790102 | 0.585624 |
|  | QIAN880104 | 0.585453 |
|  | GEIM800102 | 0.585179 |
|  | QIAN880102 | 0.585161 |
|  | GOLD730101 | 0.584536 |
|  | BEGF750102 | 0.584515 |
|  | QIAN880120 | 0.58442 |
|  | MUNV940105 | 0.584162 |
|  | KUMS000104 | 0.584098 |
|  | KARP850102 | 0.583841 |
|  | KUMS000103 | 0.583772 |
|  | CEDJ970103 | 0.583528 |
|  | GEOR030108 | 0.583491 |
|  | GEOR030104 | 0.583457 |
|  | AURR980112 | 0.583368 |
|  | JUNJ780101 | 0.583322 |
|  | LEWP710101 | 0.583242 |
|  | RACS820108 | 0.583157 |
|  | PALJ810113 | 0.583099 |
|  | RICJ880111 | 0.582996 |
|  | JUKT750101 | 0.582984 |
|  | COSI940101 | 0.582983 |
|  | GEOR030107 | 0.582955 |
|  | PALJ810115 | 0.582557 |
|  | FAUJ880110 | 0.582523 |
|  | PALJ810102 | 0.582483 |
|  | QIAN880119 | 0.582296 |
|  | BEGF750103 | 0.582182 |
|  | GEOR030109 | 0.582112 |
|  | QIAN880111 | 0.582059 |
|  | OOBM850102 | 0.582039 |
|  | ROBB760101 | 0.582007 |
|  | OOBM850101 | 0.581832 |
|  | KANM800101 | 0.581774 |
|  | TANS770104 | 0.581669 |
|  | GEIM800109 | 0.581627 |
|  | OOBM850104 | 0.581615 |
|  | DAYM780101 | 0.58159 |
|  | AURR980115 | 0.581552 |
|  | QIAN880124 | 0.581524 |
|  | PTIO830101 | 0.581511 |
|  | NOZY710101 | 0.581349 |
|  | QIAN880114 | 0.581215 |
|  | QIAN880123 | 0.581184 |
|  | QIAN880130 | 0.581125 |
|  | LEVM780104 | 0.580917 |
|  | AURR980109 | 0.58075 |
|  | GEOR030102 | 0.580325 |
|  | CHOP780201 | 0.580205 |
|  | QIAN880105 | 0.580069 |
|  | FAUJ880108 | 0.579746 |
|  | FASG760104 | 0.579606 |
|  | FUKS010109 | 0.579545 |
|  | AURR980107 | 0.579241 |
|  | LEVM780101 | 0.579077 |
|  | PRAM900102 | 0.579077 |
|  | ROBB790101 | 0.579003 |
|  | MUNV940101 | 0.579003 |
|  | QIAN880103 | 0.578809 |
|  | RICJ880116 | 0.578797 |
|  | NAKH900101 | 0.578726 |
|  | PALJ810107 | 0.578479 |
|  | VELV850101 | 0.578396 |
|  | QIAN880118 | 0.577603 |
|  | NAGK730103 | 0.577601 |
|  | PALJ810101 | 0.577463 |
|  | QIAN880138 | 0.577453 |
|  | CRAJ730101 | 0.577253 |
|  | TANS770105 | 0.577108 |
|  | FAUJ880113 | 0.576812 |
|  | QIAN880108 | 0.576605 |
|  | AURR980108 | 0.576426 |
|  | RICJ880114 | 0.576178 |
|  | QIAN880137 | 0.576076 |
|  | NAKH920101 | 0.57575 |
|  | FUKS010110 | 0.5751 |
|  | GEOR030101 | 0.575034 |
|  | RACS820112 | 0.574919 |
|  | QIAN880110 | 0.574819 |
|  | KANM800103 | 0.57376 |
|  | RICJ880110 | 0.572675 |
|  | SUEM840102 | 0.572491 |
|  | QIAN880116 | 0.572403 |
|  | RACS820107 | 0.572292 |
|  | QIAN880136 | 0.572021 |
|  | NADH010107 | 0.571981 |
|  | CHAM830102 | 0.571548 |
|  | TANS770102 | 0.571481 |
|  | RICJ880101 | 0.57148 |
|  | RICJ880102 | 0.57148 |
|  | BUNA790101 | 0.570317 |
|  | RICJ880107 | 0.570179 |
|  | GEOR030103 | 0.570061 |
|  | CEDJ970101 | 0.570058 |
|  | NAKH920103 | 0.56981 |
|  | AURR980103 | 0.569645 |
|  | NAGK730101 | 0.569433 |
|  | QIAN880128 | 0.568722 |
|  | ROBB760111 | 0.568502 |
|  | WERD780102 | 0.568313 |
|  | QIAN880126 | 0.568017 |
|  | AURR980101 | 0.567113 |
|  | CHAM830103 | 0.564974 |
|  | NAKH920104 | 0.564965 |
|  | FUKS010112 | 0.564097 |
|  | ROBB760107 | 0.564056 |
|  | FAUJ880105 | 0.563299 |
|  | JOND920101 | 0.562937 |
|  | TANS770108 | 0.562143 |
|  | RACS820109 | 0.561164 |
|  | QIAN880109 | 0.560778 |
|  | RICJ880117 | 0.560669 |
|  | RICJ880106 | 0.559872 |
|  | VENT840101 | 0.559249 |
|  | ROBB760109 | 0.558872 |
|  | CEDJ970102 | 0.558633 |
|  | CEDJ970104 | 0.550543 |
|  | CHAM830105 | 0.550389 |
|  | CHAM830104 | 0.550325 |
|  | BUNA790103 | 0.548925 |
|  | CHAM830108 | 0.536722 |
|  | CHAM830107 | 0.522538 |
|  | FAUJ880112 | 0.511703 |

*Note*: The AUC values were calculated based on the ten-fold cross-validation dataset (Figure S1 and Table S1). The physicochemical properties with an AUC value of > 0.7 for the prediction of Kmal sites were highlighted in red.
