# Supplementary material for: Integration of A Deep Learning Classifier with A Random Forest Approach for Predicting Malonylation Sites
Source: Genomics Proteomics Bioinformatics. 2019 Jan 11;16(6):451–9. doi: 10.1016/j.gpb.2018.08.004 (PMC6411950; doi:10.1016/j.gpb.2018.08.004)
Supplement: Supplementary data 7 [file mmc7.docx]

**Table S3** **Performance comparison of the different classifiers for Kmal prediction**

|  | **Classifier** | **Accuracy (%)** | **Sensitivity (%)** | **Specificity (%)** | **MCC** | **AUC** | **AUC01** |
| --- | --- | --- | --- | --- | --- | --- | --- |
| Ten-fold  cross-validation | RF_EAAC_ | **87.62** | **30.53** | **90.76** | **0.160** | **0.744** | **0.020** |
|  | RF_AAindex_ | 86.72 | 30.81 | 90.03 | 0.152 | 0.735 | 0.019 |
|  | RF_AAC_ | 87.14 | 23.67 | 90.88 | 0.112 | 0.680 | 0.015 |
|  | RF_BLOSUM62_ | 86.69 | 21.95 | 90.52 | 0.095 | 0.651 | 0.015 |
|  | RF_CKSAAP_ | 87.45 | 29.92 | 90.84 | 0.157 | 0.676 | 0.020 |
|  | RF_Binary_ | 86.66 | 21.69 | 90.44 | 0.092 | 0.649 | 0.015 |
|  | RF_Z-scales_ | 86.72 | 23.10 | 90.47 | 0.103 | 0.670 | 0.015 |
|  | CNN_WE_ | 87.97 | 36.79 | 90.99 | 0.207 | 0.809 | 0.022 |
|  | LSTM_one-hot_ | 87.27 | 41.15 | 90.00 | 0.222 | 0.813 | 0.023 |
|  | LSTM_WE_ | **88.13** | **39.79** | **90.98** | **0.228** | **0.827** | **0.025** |
|  | LEMP | **88.12** | **41.70** | **90.99** | **0.246** | **0.827** | **0.026** |
| Independent test | RF_EAAC_ | **86.83** | **31.90** | **90.08** | **0.160** | **0.739** | **0.020** |
|  | RF_AAindex_ | 86.85 | 28.48 | 90.47 | 0.144 | 0.727 | 0.016 |
|  | RF_AAC_ | 85.92 | 19.69 | 90.04 | 0.075 | 0.656 | 0.011 |
|  | RF_BLOSUM62_ | 86.54 | 22.23 | 90.34 | 0.095 | 0.651 | 0.014 |
|  | RF_CKSAAP_ | 86.74 | 31.35 | 90.01 | 0.156 | 0.658 | 0.011 |
|  | RF_Binary_ | 86.83 | 20.96 | 90.72 | 0.090 | 0.647 | 0.013 |
|  | RF_Z-scales_ | 86.73 | 23.24 | 90.48 | 0.104 | 0.667 | 0.013 |
|  | CNN_WE_ | 87.04 | 39.48 | 90.00 | 0.215 | 0.807 | 0.022 |
|  | LSTM_one-hot_ | 87.17 | 41.68 | 90.00 | 0.230 | 0.811 | 0.023 |
|  | LSTM_WE_ | **88.00** | **39.84** | **90.00** | **0.233** | **0.824** | **0.024** |
|  | LEMP | **87.30** | **43.79** | **90.00** | **0.244** | **0.827** | **0.026** |

*Note*: The datasets for ten-fold cross-validation and an independent test were derived from experimentally-verified Kmal-containing peptides. The performance of Kmal prediction was tested by combining different algorithms and encoding schemes. RF, random forest; LEMP, LSTM-based Ensemble Malonylation Predictor; MCC, Matthew’s correlation coefficient; AUC, area under the receiver operating characteristic; AUC01, AUC at a false positive rate below 10% (*i.e.*, specificity > 90%).
